# Functionalization of PEG-AgNPs Hybrid Material to Alleviate Biofouling Tendency of Polyethersulfone Membrane

**DOI:** 10.3390/polym14091908

**Published:** 2022-05-07

**Authors:** Afrillia Fahrina, Nasrul Arahman, Sri Aprilia, Muhammad Roil Bilad, Silmina Silmina, Widia Puspita Sari, Indah Maulana Sari, Poernomo Gunawan, Mehmet Emin Pasaoglu, Vahid Vatanpour, Ismail Koyuncu, Saeid Rajabzadeh

**Affiliations:** 1 Doctoral Program, School of Engineering, Post Graduate Program, Universitas Syiah Kuala, Jl. Syeh A. Rauf, No. 7, Banda Aceh 23111, Indonesia; afrilliafahrina26@gmail.com; 2 Department of Chemical Engineering, Universitas Syiah Kuala, Jl. Syeh A. Rauf, No. 7, Banda Aceh 23111, Indonesia; sriaprilia@unsyiah.ac.id (S.A.); minaelsilmina@gmail.com (S.S.); widiapuspita.scione@gmail.com (W.P.S.); indahmaulanasari2000@gmail.com (I.M.S.); 3 Graduate School of Environmental Management, Universitas Syiah Kuala, Jl. Tgk. Chik Pante Kulu No. 5, Banda Aceh 23111, Indonesia; 4 Research Center for Environmental and Natural Resources, Universitas Syiah Kuala, Jl. Hamzah Fansuri, No. 4, Banda Aceh 23111, Indonesia; 5 Atsiri Research Center, PUI, Universitas Syiah Kuala, Jl. Syeh A Rauf, No. 5, Banda Aceh 23111, Indonesia; 6 Faculty of Integrated Technologies, Universiti Brunei Darussalam, Jalan Tungku Link, Gadong BE 1410, Brunei Darussalam; roil.bilad@ubd.edu.bn; 7 School of Chemical and Biomedical Engineering, Nanyang Technological, University Singapore, Singapore 627833, Singapore; pgunawan@ntu.edu.sg; 8 National Research Center on Membrane Technologies, Istanbul Technical University, Maslak, Istanbul 34469, Turkey; mpasaoglu@itu.edu.tr (M.E.P.); vahidvatanpoor@yahoo.com (V.V.); koyuncu@itu.edu.tr (I.K.); 9 Department of Environmental Engineering, Istanbul Technical University, Maslak, Istanbul 34469, Turkey; 10 Department of Applied Chemistry, Faculty of Chemistry, Kharazmi University, Tehran 15719-14911, Iran; 11 Research Center for Membrane and Film Technology, Department of Chemical Science and Engineering, Kobe University, Rokkodai-Cho 1-1, Nadaku, Kobe 657-0000, Japan; rajabzadehk@people.kobe-u.ac.jp

**Keywords:** hybrid materials, anti-bacterial, membranes, anti-biofouling

## Abstract

Membrane-based processes are a promising technology in water and wastewater treatments, to supply clean and secure water. However, during membrane filtration, biofouling phenomena severely hamper the performance, leading to permanent detrimental impacts. Moreover, regular chemical cleaning is ineffective in the long-run for overcoming biofouling, because it weakens the membrane structure. Therefore, the development of a membrane material with superior anti-biofouling performance is seen as an attractive option. Hydrophilic-anti-bacterial precursor polyethylene glycol-silver nanoparticles (PEG-AgNPs) were synthesized in this study, using a sol-gel method, to mitigate biofouling on the polyethersulfone (PES) membrane surface. The functionalization of the PEG-AgNP hybrid material on a PES membrane was achieved through a simple blending technique. The PES/PEG-AgNP membrane was manufactured via the non-solvent induced phase separation method. The anti-biofouling performance was experimentally measured as the flux recovery ratio (FRR) of the prepared membrane, before and after incubation in *E. coli* culture for 48 h. Nanomaterial characterization confirmed that the PEG-AgNPs had hydrophilic-anti-bacterial properties. The substantial improvements in membrane performance after adding PEG-AgNPs were evaluated in terms of the water flux and FRR after the membranes experienced biofouling. The results showed that the PEG-AgNPs significantly increased the water flux of the PES membrane, from 2.87 L·m^−2^·h^−1^ to 172.84 L·m^−2^·h^−1^. The anti-biofouling performance of the PES pristine membrane used as a benchmark showed only 1% FRR, due to severe biofouling. In contrast, the incorporation of PEG-AgNPs in the PES membrane decreased live bacteria by 98%. It enhanced the FRR of anti-biofouling up to 79%, higher than the PES/PEG and PES/Ag membranes.

## 1. Introduction

The escalation of clean water demand increases the need to employ a reliable membrane-based process for drinking water and wastewater treatments [1,2]. The pressure-driven membrane processes, such as ultrafiltration (UF), nanofiltration (NF), and reverse osmosis (RO), offer a high efficiency of separation and purification, with no or minimum input of chemicals. Polyethersulfone (PES), polyvinylidene fluoride (PVDF), and polysulfone (PSF) polymers are frequently used as membrane materials, due to their high resistance to hydrolytic, oxidative, thermal, and chemicals disturbance [3,4,5]. However, due to their low surface energy and high hydrophobicity, fouling phenomena that disrupt filtration performance using those pristine polymeric membranes are inevitable [6,7]. Various foulants in the feed solutions, such as bacteria, polysaccharides, proteins, and humic acids, attach to membrane pores [8]; hence, lowering the process throughput.

Membrane fouling induced by microorganism growth on the membrane surface is called biofouling. It worsens the membrane performance by a higher magnitude than organic, inorganic, and colloids [9,10]. Formation of biofouling is started when microbial cells are first deposited on the surface induced by drag and hydraulic conditions, Brownian force, and gravity, and then adhere to the membrane surface by the action of interface energy [11]. Through quorum-sensing signal molecules for bacterial cell-to-cell communication, the bacteria collectively grow and proliferate, forming biofilm [10]. The biofilm structure is finally strengthened by extracellular polymeric substances (EPS) containing lipids, proteins, polysaccharides, and nucleic acids, as well as bacterial bio-secretion, and causes biologically irreversible fouling [7,10,12,13]. During membrane operation, the irreversible biofouling reduces the permeability significantly, increases energy consumption, lowers filterability, and necessitates regular cleanings [11,14,15]. In this case, chemical cleanings are employed to eliminate the biofilm, because a physical cleaning is only practical to remove loosely-bound materials (reversible fouling) [16]. Consequently, the chemical cleanings gradually weaken the membrane integrity and shorten its life span [12,17].

Research on membrane biofouling mitigation has recently been reported, regarding improving the anti-bacterial properties of the membrane by preventing attachment, quorum quenching, and killing bacteria [11,18,19]. Anti-attachment involves modifying the physicochemical properties of the membrane surface, such as the hydrophobicity, electrostatic charge, and surface roughness [10,20,21]. Quorum sensing inhibition or quorum quenching methods interrupt cell to cell communication, to prevent biofilm formation [11,22]. The killing strategy targets killing bacteria around the membrane by attacking their functional cellular system [23,24]. Various organics or inorganic materials possess anti-bacterial properties, such as chitosan, quaternary ammonium compound, silver (Ag), graphene oxide, etc. They are commonly employed as an additive in membrane fabrication by blending, grafting, or coating methods [25,26,27,28,29,30].

The Kirby Bauer disc diffusion method is frequently used to confirm the membrane’s anti-bacterial properties by showing the bacterial inhibition zone [31,32]. Some researchers have also applied the total plate counting method (TPC) to calculate the number of live bacterial colonies [7,33]. Advanced equipment, such as confocal laser scanning microscopy or scanning electron microscopy (SEM), is another approach to proving the anti-bacterial properties, by visualizing the presence of bacterial adhesion on the membrane surface [14,34]. However, these approaches are only sufficient to prove membrane anti-bacterial characteristics and do not comprehensively confirm the anti-biofouling of a membrane experimentally. 

The present work proposed hydrophilic-antibacterial additives of polyethylene glycol-silver nanoparticles (PEG-AgNPs), prepared by a simple sol-gel method. Ag particles were chosen since they possess supreme anti-bacterial properties, with killing effects and a low cytotoxicity [35,36]. Incorporating Ag particles and PEG polymers into Janus nanoparticles was shown to improve the physicochemical properties of the ultimate materials thanks to their PEG biocompatibility, high water solubility, and nontoxicity [35,37,38]. The PEG-AgNPs were chemically functionalized and loaded into the PES membrane via a simple blending method, to improve the anti-biofouling performance. The AgNPs were aimed at preventing the biofilm formation derived from bacteria proliferation, while hydroxyl groups of the PEG polymer chain could weaken the EPS–membrane attachment. The anti-biofouling of the membrane was evaluated experimentally, in terms of the flux recovery ratio. At the same time, the irreversible biofouling resistance was measured as flux decline after incubation in an Gram-negative bacteria, *Escherichia coli* culture. 

## 2. Materials and Methods

### 2.1. Materials

PES (Ultrason E6020P) was purchased from BASF (Lemförde, Germany). *N*-Methyl pyrrolidone (NMP) with analytical grade purity of 99.9% was purchased from Sigma Aldrich, Singapore. Polyethylene glycol (PEG, 4 kDa) and silver nitrate (AgNO_3_) were purchased from Merck KGAa (Darmstadt, Germany). The other chemicals and agents employed in this study were *Escherichia coli* (ATCC 25922), Mueller-Hinton agar (Oxoid, Basingtoke, England), Ciprofloxacin (Oxoid, Basingtoke, England), and distilled water.

### 2.2. Synthesis of PEG-AgNPs Hybrid Materials

A sol-gel process was applied to synthesize PEG-AgNP hybrid particles. In 500 mL of distilled water, a 25% PEG solution was prepared and agitated at room temperature until homogenous. Into the solution, 20 mL of AgNO_3_ with a concentration of 2% was added, dropwise. The solution was stirred vigorously at 80 °C for 4 h and incubated in a darkroom for 72 h, to assess its stability. Then, the PEG–AgNPs hybrid solution was frozen at −66.1 °C in an ultralow temperature freezer. Finally, a freeze dryer (Lyovaporator L-300) was used for 70 h to sublimate the PEG-AgNPs ices to a dried powder at −104.4 °C and 10.0 mbar. The physical-chemical and biological properties of the PEG–AgNPs were then analyzed in terms of their hydrophilicity and anti-bacterial properties.

### 2.3. Preparation of PES/PEG-AgNPs Membrane

**Figure 1:**
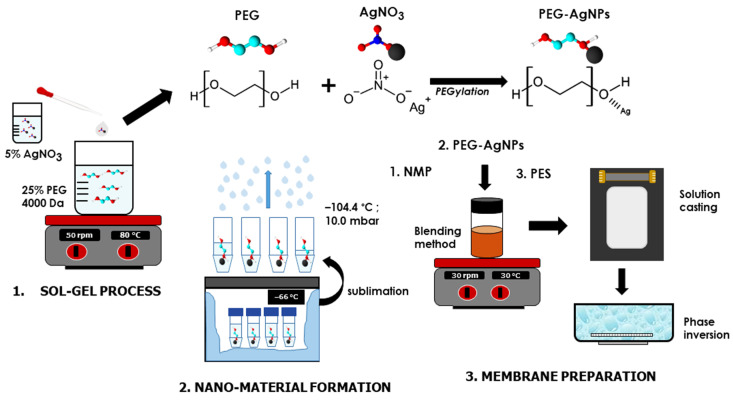
Comprehensive schematic diagram of additive and membrane preparation.

The non-solvent induced phase separation (NIPS) approach was applied to fabricate a flat-sheet membrane at ambient temperature. Homogeneous dope solutions (listed in Table 1) were initially prepared by dissolving a precise quantity of PEG-AgNPs in NMP, followed by the addition of PES under continuous stirring. Pure PEG and AgNO_3_ incorporated PES membranes were also employed as a non-hybrid additive. The transparent and bubble-free dope solutions were then cast onto a flat-squared glass with an air gap of 300 µm using an adjustable applicator. Subsequently, the obtained polymer films were immersed in a batch containing distilled water for 5 min, to allow the phase inversion to form a solid membrane matrix. The resulting membranes were finally stored in wet conditions for further analysis and used for the filtration tests. Figure 1 illustrates the complete preparation method employed in this study.

### 2.4. Characterization of the PEG-AgNPs Hybrid Material

A Nicolet™ iS50 FTIR Spectrometer (Thermo Fisher Scientific, Rockford, IL, USA), with the ATR method, was applied to determine the chemical bonding of the hybrid material. The particle size and the zeta potential of PEG-AgNPs were measured as hydrodynamic diameter by applying the dynamic light scattering method using PSA Horiba-Sz 100z (Horiba, Ltd., Kyoto, Japan). The morphological structure of the dried PEG-AgNPs was analyzed using transmission electron microscopy (TEM, JEOL JEM-1400, Tokyo, Japan), while further identification of the particle size distribution was processed using *ImageJ* software (1.53e, USA). A bacterial assay against *Escherichia coli* was confirmed by Kirby Bauer disc diffusion, with ciprofloxacin used as a positive control (control +) and distilled water as a negative control (control −). The particle size and the zeta potential of the PEG-AgNPs were measured as the hydrodynamic diameter, by applying a dynamic light scattering method using PSA Horiba-Sz 10.

### 2.5. Characterization and Selectivity Performance of PES/PEG-AgNPs Membranes

A Nicolet™ iS50 Fourier Transform Infrared (FTIR) Spectrometer (Thermo Fisher Scientific, Rockford, IL, USA) was utilized to confirm the chemical groups in the membrane surface, based on the ATR method. The morphological structure of the cross-section membranes was observed by SEM (Philips-XL30 SFEG, York Probe Sources Ltd., UK) in ESEM mode, using a gold-palladium (Au-Pd) coating. The elemental composition of membranes was detected and mapped with an energy-dispersive spectrometer (EDS, JEOL JED-2300, Tokyo, Japan). *ImageJ* software (1.53e, USA) was utilized to determine the overall porosity of the membrane. Furthermore, the Guerout–Elford–Ferry Equation (1) was applied to estimate the membrane pore size (*r_m_*):

(1) $$r_{m}=\sqrt{\frac{\left(2.9-1.7\varepsilon \right)8ƞlQ}{\varepsilon A\Delta P}}$$

where *η* is the water viscosity (8.9 × 10^−4^ Pa·s), *Q* is the rate of permeate volume time (cm^3^·s^−1^), and Δ*P* is the driven pressure (2 × 10^5^ Pa).

The water uptake rate of the membranes was measured, to predict the membrane hydrophilicity. A flat membrane coupon of 2 × 2 cm^2^ was dried in a drying oven at 45 °C for 24 h, and the dried mass was weighed (*w_d_*). The dried membrane was then immersed in distilled water at 25 °C for 24 h. The specimen was wiped with tissue paper and then weighed again (*w_w_*). The gravimetric method measurements were done in triplicate. The water uptake of the membrane was defined using Equation (2):

(2) $$Water\;uptake\;rate=\frac{w_{w}-w_{d}}{\;\;w_{w\;\;}}\times 100\%$$

where *w_w_* and *w_d_* are the wet and dried membrane weight (g), respectively, *ε* is the membrane porosity, *A* is the membrane sample surface area (cm^2^), *l* is the membrane sample thickness (cm), and *ρ_w_* is the density of water (g·cm^−3^).

The pure water flux was measured using a bench-scale cross-flow filtration system. A flat-sheet membrane with an effective area of 9.075 cm^2^ was mounted into the filtration cell. A peristaltic pump and a pressure controller were used to apply a constant feed flow rate of 14 mL·min^−1^ under a transmembrane pressure of 0.2 MPa. The steady water flux ($J_{w}$) was calculated using Equation (3):

(3) $$J_{w}=1-\frac{V_{p}}{A_{m}t_{f}}$$

where $J_{w}$ is the water flux (L·m^−2^·h^−1^); $V_{p}$ is permeate volume (L); $A_{m}$ is the effective membrane area (m^2^); and $t_{f}$ is permeate collecting time (h).

Moreover, the filtration performance of the membranes was tested using a humic acid (HA) solution of 10 mg·L^−1^ as the pollutant model. A spectrophotometer (Shimadzu UV-1800, Kyoto-Japan) was used to measure the initial and final concentrations of HA. The rejection value ($R_{m}$) was calculated according to Equation (4).

(4) $$R_{m}=\left(1-\frac{C_{p}}{C_{f}}\right)\times 100\%$$

where *R_m_* is the rejection value of the HA solution (%), and *C_p_* and *C_f_* are the HA concentration in the permeate and feed tank (mg·L^−1^), respectively.

### 2.6. Evaluation of Membrane Anti-Bacterial and Anti-Biofouling Performance

In this study, a pre-analysis of the membrane in preventing biofouling was conducted by investigating the anti-bacterial activity. All membrane samples were first sterilized by UV radiation (22 W, SUV-16 254 nm AS ONE, Japan) for 30 min. *E. coli* (ATCC 25922) solution was prepared with a high concentration of 0.5 McFarland or 1.5 × 10^8^ colony forming units (CFU·mL^−1^) and swabbed onto Mueller–Hinton agar (MHA) media using a sterile cotton. Then, the sterile membrane samples were put on the MHA surface and incubated for 24 h at 37 °C. The number of colonies that grew on the membrane surface was calculated using a colony counter under a magnifying glass. 

Next, the anti-biofouling of the membranes was experimentally evaluated using a cross-flow module. Initially, the steady water flux of the membrane (*J_w_*_1_) was measured. Applying the same step as above, the membrane samples were incubated in *E. coli* culture (1.5 × 10^8^ CFU·mL^−1^) for 48 h, to allow the biofilm formation. After biofouling exposure, the membrane was physically cleaned by immersing it in distilled water to remove the reversible biofouling. Subsequently, the cleaned membrane was re-evaluated by measuring the constant water flux (*J_w_*_2)_. The parameter of anti-biofouling performance was represented as the flux recovery ratio (FRR), as defined in Equation (5):

(5) $$FRR=\frac{J_{w2}}{J_{w1}}\times 100\%$$

While the irreversible biofouling resistance was measured as flux decline (FD) in Equation (6):

(6) $$FD=\left(1-\frac{J_{w2}}{J_{w1}}\right)\times 100$$

## 3. Result and Discussions

### 3.1. Characterization of PEG-AgNPs

PEGylation was performed to facilitate the chemical bonding of PEG and Ag^+^. The bonding was expected due to the Van der Waals forces that occurred from the interaction of polar ions in the solution. The Ag^+^ interacted with the negative charge of oxygen in the hydroxyl group (–OH) of PEG to form the nucleus (Ag (PEG)) [39]. In this case, the PEG played a role as a reducing agent of Ag^+^ to Ag. This occurred through the oxidation of hydroxyl groups in the PEG chain to aldehyde groups (CH_2_CH_2_OH → CH_2_CHO) [40,41].

**Figure 2:**
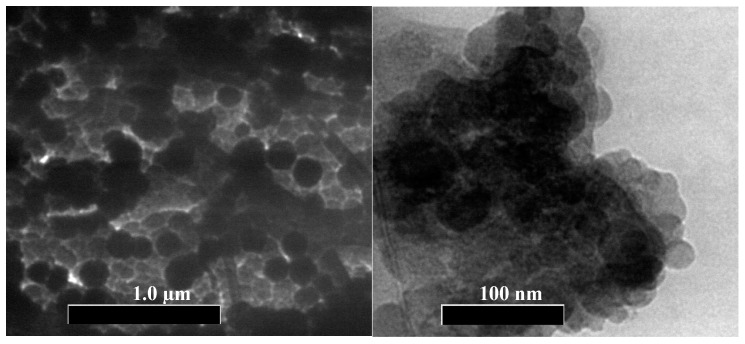
TEM image of the PEG-AgNPs under different magnifications.

**Figure 3:**
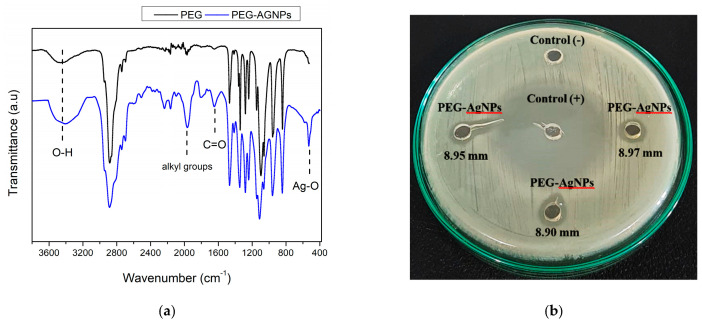
FTIR spectra (**a**) and anti-bacterial performance (**b**) of the PEG-AgNPs.

The TEM micrographs in Figure 2 depict the morphology of PEG-AgNPS. A uniform spherical shape in the range of 10–24 nm was observed. Table 2 shows that the modus of particles size distribution diameter processed by *image J* was 16.4 nm. The hydrodynamic diameter measured by DLS method showed an average modus value of 103.8 nm, with a cumulative size range of 72.6–128.0 nm (Table 2). The zeta potential value of −17 mV indicated that the PEG-AgNPs particles tended to agglomerate, generating a larger diameter in an aqueous solution. The transmittance spectra of the PEG and PEG-AgNPs in a range of 4000–400 cm^−1^ are shown in Figure 3. The desired O–H group was presented in the PEG-AgNPs and PEG spectra, at the broad coverage area of 3300–3600 cm^−1^. C–H stretching in the aliphatic group appeared in the PEG and the PEG-AgNPs at a wavenumber of 2880 cm^−1^. There is a peak at 2200 cm^−1^ in PEG-AgNPs spectra that likely indicates the presence of C≡C. Some similar bands are also founded in both spectra, such as the bands at a wavenumber of 1466 cm^−1^ and 1340 cm^−1^, which correspond to C–H bending vibrations groups, and 1279 cm^−1^ to 1146 cm^−1^, attributed to C–O, C–O–C stretching [42]. A sharp peak at 1095 cm^−1^ was attributed to C–C–O and C–C–H. In the PEG-AgNPs, an increase in the intensity at 1654 cm^−1^ was related to the C=O, which implies a weak coordination chemical bonding between C=O and Ag. The substituted alkyl group and allene groups appeared at 1956^−1^ cm, which also might be the products of PEG oxidation [43]. Furthermore, the presence of Ag-O bonding was detected at 530 cm^−1^ in the PEG-AgNPs spectra, which is attributed to the banding of Ag^+^ with oxygen from hydroxyl groups of the PEG chains [42]. 

The PEG-AgNP’s anti-bacterial property was demonstrated through qualitative and quantitative analyses. Figure 3 shows the anti-bacterial assays of PEG-AgNPs against *E. coli* using Kirby Bauer disc diffusion methods. A clear area in the circle around the PEG-AgNPs and ciprofloxacin was used as the positive control (control +). The clear zone implied a bacterial inhibition zone, where there was no bacterial growth. In contrast, no inhibition zone (clear space) was found on the negative control (control −). Ciprofloxacin had the most robust anti-bacterial characteristics, with a 32.68 mm inhibition zone, while the PEG-AgNPs showed an anti-bacterial spot of 3.94 mm.

Since *E. coli* is negatively charged [44], the Ag^+^ most likely adhered to the cell membrane, due to the electrostatic force. Owing to the nano-size structure of PEG-AgNPs, the Ag^+^ could easily infiltrate the bacterial cell and reach the cytoplasm. The interaction of Ag^+^, between the cellular structures and the biomolecules of *E. coli,* led to some bacterial malfunctions; they could suppress the intracellular biological system and inactivate the respiratory chain [45]. The effect of Ag^+^ was lethal to *E. coli.*

### 3.2. Characteristics of the PES/PEG-AgNPs Membrane

#### 3.2.1. Chemical Groups and Elemental Composition

**Figure 4:**
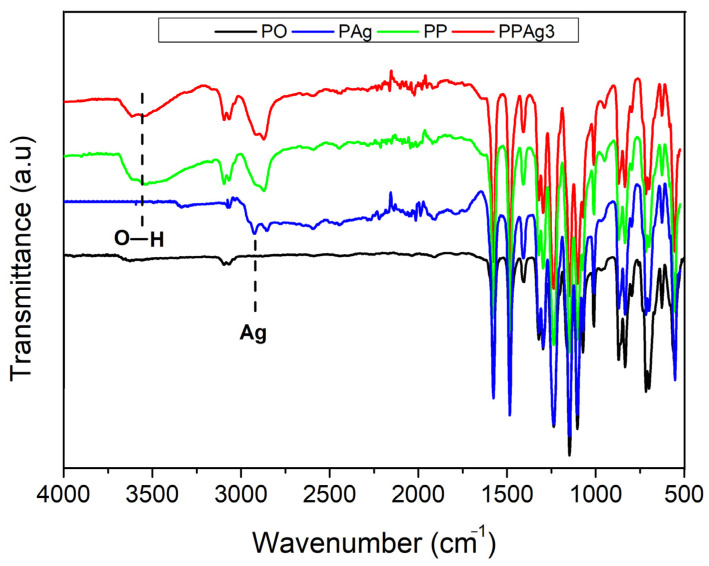
FTIR analysis of the membranes’ surface.

Figure 4 shows the membrane surfaces ATR-FTIR spectra used for identifying chemical bands. All spectrums exhibited the PES membrane type, consisting of asymmetric and symmetric stretching vibrations of the S=O group at 1237 cm^−1^ and 1147 cm^−1^. The bending oscillation of the PES aromatic ring was also observed at peaks of 1484 cm^−1^ and 1576 cm^−1^ [46]. An intense C–O strain was present at 1077–1100 cm^−1^ [47]. 

After the incorporation of additives, some new bands appeared. In the PES/PEG and the PES/PEG-AgNPs membranes, the O–H groups appeared at approximately 3500 cm^−1^. This hydroxyl group emphasized the hydrophilicity of the PEG-AgNPs-incorporated PES membrane. Similar stretching bands at 2840 and 3000 cm^−1^ were present in both membranes, as indicated by the C–H bonds from the alkane group of PEG [5]. The low peak at 2924 cm^−1^ in the spectra of the PES/Ag membrane might confirmed the presence of Ag [36]. However, the Ag band did not appear on the PES/PEG-AgNPs membrane, possibly due to the low Ag concentration after the hybridization. Therefore, a EDS analysis was further conducted to confirm its presence.

**Figure 5:**
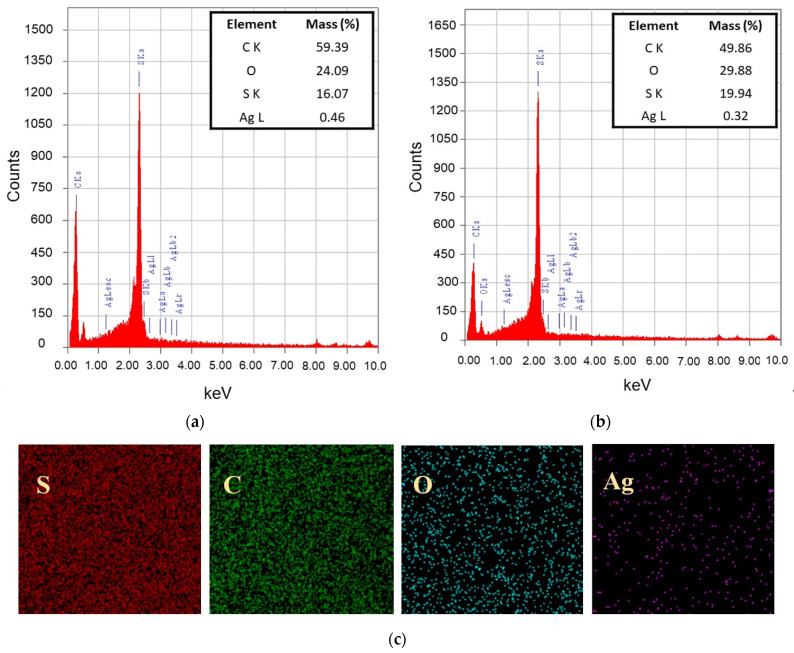
EDS elemental identification for Pag (**a**) and PPAg3 (**b**) membranes; topology of PPAg3 membrane surface (**c**).

Figure 5a,b show the elemental composition of PES/Ag and PES/PEG-AgNPs-modified membranes consisting of C, O, S, and Ag elements. The C, O, and S peaks are naturally derived from PES as the primary building blocks of the membrane. The enhancement of O element composition in the PES/PEG-AgNPs was attributed to OH groups from PEG. An Ag peak was observed in the spectrum at 3 keV in both the PES/Ag and PES/PEG-AgNPs membranes. The low composition of Ag in the PES/PEG-AgNPs membrane was due to PEG and Ag’s hybridization, which reduced the total fraction of Ag in the nanoparticles. It can also be attributed to the leaching out of nanoparticles from the dope film to the non-solvent during the NIPS, due to the hydrophilic nature of the PEG-AgNPs. The elemental mappings in Figure 5c confirmed the uniform distribution of Ag on the PES/PEG-AgNPs membrane surface.

#### 3.2.2. Membrane Morphological Structure

**Figure 6:**
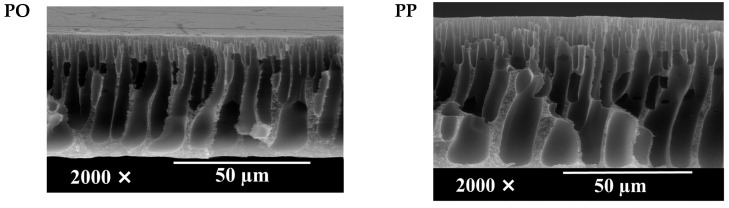

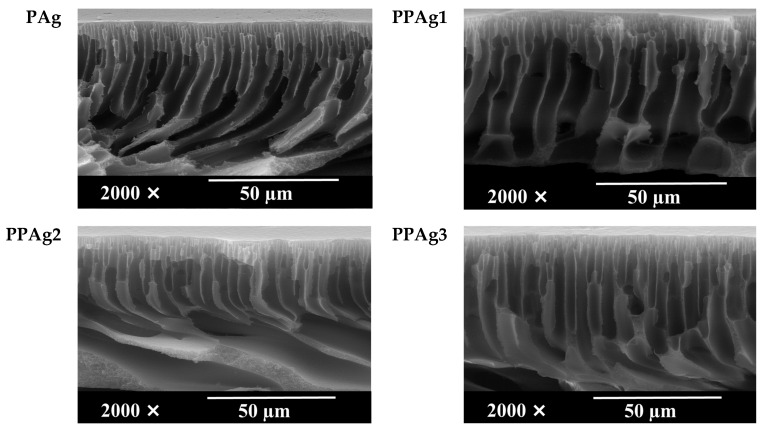
SEM images of membrane morphological structure.

Figure 6 shows an asymmetric structure arranged as a skin top layer, supported by a finger-like void. The kinetic aspect governed the final membrane structure during the phase inversion [48]. The good affinity between NMP and water encouraged the rapid outflow of NMP (from the cast film to the bulk water in the coagulation bath) and water inflow (from the coagulation bath into the polymer film). The instantaneous de-mixing resulted in the finger-like structure [49]. However, the pore size was also affected by the type, size, and amount of additives, which affect the dope solution chemistry and viscosity [50].

As shown in Figure 6, the hydrophilic PEG additive’s high molecular weight resulted in a larger finger size in the PES membrane (C1) compared to the AgNO_3_ additive (C2). The hydrophilicity and the size of the additive also affected the final porosity. Conversion to a Janus-type of additive in the form of PEG-AgNPs altered the porosity. The addition of 3% PEG-AgNPs resulted in the largest size of the macrovoid. The hydrophilic nature of the PEG-AgNPs and the large surface area of the nanoparticles improved the affinity of the membrane solution towards the water, leading to fast diffusivity during the NIPS. In addition, certain amounts of the PEG-AgNPs were evacuated from the film, leaving spaces occupied by water, which later turned into the macrovoid. However, increasing the PEG-AgNPs loadings to 5 and 7 wt% in Q3 and Q4 reduced the finger-void diameter. Higher PEG-AgNPs loadings led to higher dope solution viscosity, inhibiting the mass transfer or delaying de-mixing as a kinetic hindrance during the NIPS.

#### 3.2.3. Membrane Porosity Analysis, Water Uptake Rate, and Water Flux

**Figure 7:**
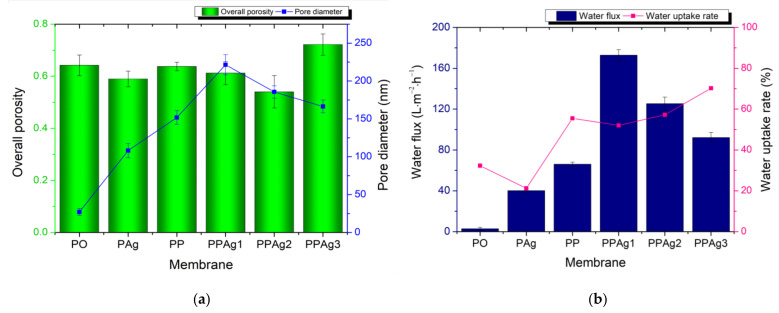
Membrane porosity and pore diameter (**a**); water uptake rate and clean water flux (**b**).

Figure 7 shows the porosity and the pore size of the membrane samples. They had a varying overall porosity, from 0.540 to 0.722. The presence of additive in the PES membrane significantly increased the average pore diameter of the membrane. The addition of 7 wt% PEG-AgNPs produced the highest membrane porosity. However, the increment of the PEG-AgNP concentration gradually reduced the membrane pore size, as confirmed by the SEM photographs in Figure 6. 

The trend of pure water flux in Figure 7 agrees with the pore size graph. It shows that the water permeation strongly correlated with the membrane morphology and the pore diameter. Due to the smallest pore size, the pristine PES exhibited the lowest water flux of 2.87 L·m^−2^·h^−1^. The addition of 5 wt% AgNPs and PEG improved the water flux to 40.20 L.m^−2^·h^−1^ and 66.16 L·m^−2^·h^−1^, respectively. Although the PES/Ag membrane had the lowest porosity, its larger pore size led to higher water permeation than the pristine PES membrane.

The water flux increased to 90–172 L·m^−2^·h^−1^ after loading the PEG-AgNPs additive. The presence of hydroxyl groups (–OH) on the membrane surface enhanced the affinity toward water. The loading of 3 wt% PEG-AgNPs achieved the highest increment of water flux of 172.84 L·m^−2^·h^−1^. As shown from the SEM images in Figure 6, the PPAg1 membrane had the largest size of finger-void. Furthermore, Figure 7 confirmed that the PPAg1 membrane had the largest pores, facilitating water permeation. However, increasing the PEG-AgNPs loading to 5–7 wt% gradually decreased the water flux, due to the smaller pore sizes.

Figure 7 also shows the membrane water absorption ability. The PEG incorporated PES membranes had a higher water uptake rate. The increase of PEG-AgNPs membrane concentration linearly improved the water uptake, most likely due to the increment of hydroxyl groups. 

### 3.3. Membrane Selectivity Performance

**Figure 8:**
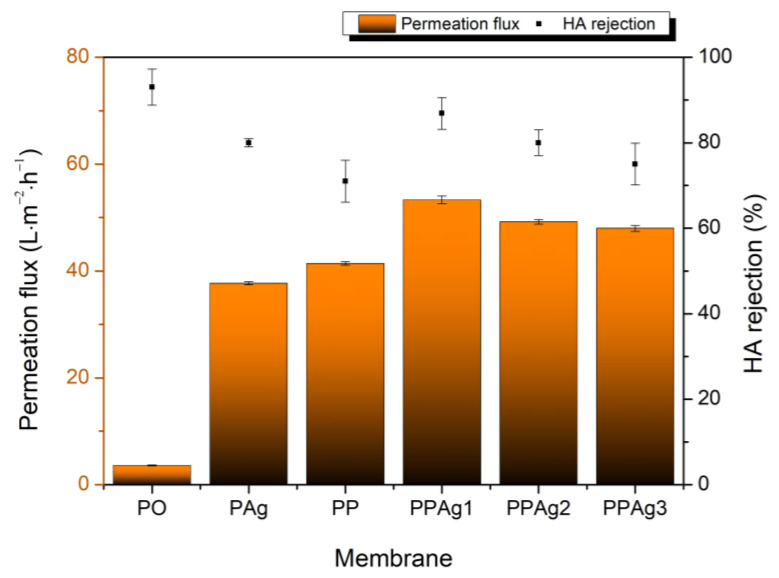
Membrane selectivity performance.

Figure 8 presents the membrane retention performance of the HA solution. The pristine PES membrane showed the best retention value of 93%, but resulted in the lowest permeation of 3.62 L·m^−2^·h^−1^. The incorporation of additive significantly increased the permeation flux but lowered the HA rejection, due to the increment of the porosity and pore size distribution (see Figure 7). PPAg1 promoted the highest permeation, with an optimal retention value of 86.9%. However, although PPAg1 had the largest pore size, its porosity was lower than the PP and PPAg3 membrane. Therefore, both membranes showed a lower rejection value, due to their higher porosity, instead of increasing the retention value.

### 3.4. Anti-Bacterial Study and Anti-Biofouling Performance of the Membrane

**Figure 9:**
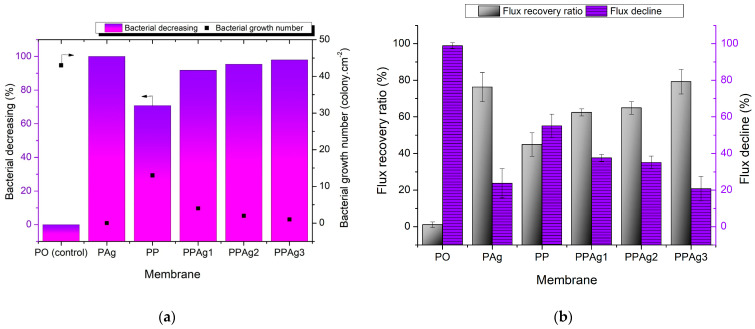
Anti-bacterial determination of the membrane surface (**a**), and anti-biofouling performance of membranes, in terms of flux recovery and flux decline (**b**).

In this study, the anti-bacterial property of the membrane was evaluated using the same method as in our previous work [33]. The pristine PES membrane was used as a control membrane to evaluate the improvement of anti-bacterial performance. Figure 9a shows the bacterial decreasing value and bacterial growth number. The highest colony count, 43 colonies·cm^−2^, was demonstrated by the pristine PES membrane. The addition of PEG in the PP membrane decreased the live bacteria by 71% to 13 colonies·cm^−2^, due to the increasing membrane hydrophilicity. It retarded the hydrophobic interaction between the bacterial cells and the membrane surfaces [20]. As expected from the PAg membrane with the loading of a high concentration of Ag^+^, no live bacteria was found on the membrane surface, which indicated a 100% bacterial decrease. Incorporating PEG-AgNPs material in the PES membrane also showed a satisfactory anti-bacterial performance. 

The increment of PEG-AgNPs concentration gradually improved the bacterial reduction value, from 92% to 98%. It was envisaged that the positive charge of Ag interacted with the negative charge of *E. coli* thanks to the electrostatic force. The contact-killing mechanisms of AgNPs against bacteria included cell membrane destruction, DNA/RNA replication disorder, and the release of toxic reactive oxygen species [51,52].

Furthermore, Figure 9b demonstrates the anti-biofouling performance of the membrane, in terms of FRR and FD. All membranes exhibited a decrease in water flux due to the biofilm formation of the *E. coli* strain. The pristine PES severe flux decline of 99% could only recover 1% of water permeation. The hydrophobic surface of the PES produced a strong attachment of irreversible biofilm, leading to critical pore blockage. The biofouling of the membrane surface may have influenced the permeate flux in two ways: (i) The foulant layer built an additional hydraulic resistance, declining the overall membrane permeability; (ii) A porous cake layer affected the membrane flux by introducing a severe concentration polarization inside the unstirred cake layer, which resulted in a severe flux loss [53].

After adding PEG, the FRR improved to 45%, thanks to the hydroxyl groups that weakened the biofilm adherence. As expected, the PES/PEG membrane (PP) showed a lower FRR than the other modified membranes, because no anti-bacterial agent prevented a strong biofilm formation and EPS secretion from the live bacteria. The PPAg1 and PPAg2 membranes showed better biofouling resistance than the PP membrane, with FRRs of 62–65%. Moreover, the PES/Ag (PAg) significantly maintained a water flux of 76%, with a 24% flux decline. The PPAg3 membrane exhibited the most satisfactory performance in the anti-biofouling test, with an FRR value of 79%. Despite the PAg membrane possessing the best anti-bacterial performance, the dead cells of bacteria clogged the membrane pores. They could not be easily removed by water flushing, due to the lack of hydrophilicity on the surface, resulting in a lower water permeation. Adding 7% PEG-AgNPs helped overcome this issue by providing a hydrophilic and killing effect on the membrane surface towards bacteria. A hydrophilic surface weakens the bacterial attachment and transformed them into reversible biofouling, which would be easily washed by a running water. At the same time, Ag^+^ prevented the attachment of bacteria and reduced the biofilm formation. 

## 4. Conclusions

This study developed a hydrophilic-antibacterial membrane additive from PEG-AgNPs using a simple sol-gel method. The effect of PEG-AgNPs in enhancing the anti-biofouling performance was investigated. The PEG-AgNP additive significantly boosted the water flux. After exposure in *E. coli* culture for 48 h, the PES/PEG-AgNPs membrane maintained an FRR of up to 79%. It showed a lower irreversible biofouling tendency, better than the pristine PES, PES/PEG, and PES/Ag membranes, thanks to the optimal anti-biofouling property imposed by the hybrid additive.

## Figures and Tables

**Table 1:** The composition of the prepared membrane solution.

Labels	Membrane Composition (wt. %)				
	PES	PEG	Ag	PEG-AgNPs	NMP
PO	18	0	0	0	82
PAg	18	0	5	0	77
PP	18	5	0	0	77
PPAg1	18	0	0	3	79
PPAg2	18	0	0	5	77
PPAg3	18	0	0	7	75

**Table 2:** The physical characteristics of the PEG-AgNPs hybrid materials.

PEG-AgNPs Characteristics	Modus Value	Units
Particle size distribution	16.4	nm
Hydrodynamic diameter	103.8	nm
Zeta potential	−17.1	mV

## Data Availability

Not applicable.
